# The Influence of Sexually Transmitted Bacteria and Human Papillomavirus on Sperm Parameters: Data from a Preliminary Study

**DOI:** 10.3390/medicina62050874

**Published:** 2026-05-03

**Authors:** Maria Samara, Eleni Thodou, Christina Messini, Efthalia Moustakli, Maria Anagnostou, Athanasios Zikopoulos, Alexandros Daponte, Ioannis Georgiou, George Anifandis

**Affiliations:** 1 Department of Pathology, Faculty of Medicine, School of Health Sciences, University of Thessaly, 41100 Larisa, Greece; masamar@uth.gr (M.S.); ethodou@uth.gr (E.T.); 2 Department of Obstetrics and Gynecology, Faculty of Medicine, School of Health Sciences, University of Thessaly, 41100 Larisa, Greece; pireaschristina@gmail.com (C.M.); managnostou@uth.gr (M.A.); daponte@uth.gr (A.D.); 3 Department of Nursing, School of Health Sciences, University of Ioannina, 4th Kilometer National Highway Str. Ioannina-Athens, 45500 Ioannina, Greece; ef.moustakli@uoi.gr; 4 Royal Cornwall Hospitals NHS Treliske Truro, Foundation Trust U.K., Truno TR1 3LJ, UK; thanzik92@gmail.com; 5 Laboratory of Medical Genetics in Clinical Practice, Faculty of Medicine, School of Health Sciences, University of Ioannina, 45110 Ioannina, Greece; igeorgio@uoi.gr; 6 Department of Histology and Embryology, Faculty of Medicine, School of Health Sciences, University of Thessaly, 41100 Larisa, Greece

**Keywords:** sperm parameters, sexually transmitted infection, HPV, infertility, semen

## Abstract

*Background and Objectives*: The microbiome plays a pivotal role in male infertility, with distinct microbial species exerting both beneficial and deleterious effects on reproductive function. Sexually transmitted bacteria and several viruses, including human papillomavirus (HPV), have been identified in semen. This cross-sectional study aimed to examine the prevalence of single and co-infections of sexually transmitted bacteria (STB)—such as *Chlamydia trachomatis, Mycoplasma* spp., and *Ureaplasma* spp.—with various HPV subtypes in Greek male partners of infertile couples and to evaluate their potential impact on sperm parameters. In addition, the possible effect of cryopreservation on the maintenance of these pathogens was assessed. *Materials and Methods*: Eighty-two semen samples were initially collected from 82 individuals undergoing routine sperm analysis. In total, 80/82 (97.6%) participants proceeded to further analysis, as 2/82 (2.4%) were excluded due to poor DNA quality. *Results*: A total of 18/80 (22.5%) sperm samples tested positive for STB, with *Ureaplasma* spp. representing the most frequently detected pathogen. Co-infection of *Ureaplasma* spp. and *Mycoplasma hominis* was observed in 4/80 (5%) samples. Twelve samples (12/80, 15%) were positive for HPV, including low-risk (LR) and high-risk (HR) types, and HPV 16 was the predominant HR genotype. Notably, a co-infection of STB and HPV was not found in our specimens. STB-positive samples demonstrated significantly higher sperm concentration and improved progressive motility compared with STB-negative samples. HPV-positive samples exhibited lower sperm volume and concentration and increased non-progressive motility compared with HPV-negative samples. Following three months of cryopreservation, LR HPV and STB were no longer detectable, whereas HR HPV types remained detectable. *Conclusions*: These preliminary findings are interesting, as they could be useful for routine screening of HPV and STB in sperm samples preserved in sperm banks and highlight the need for future research.

## 1. Introduction

Infertility is a common issue among couples of reproductive age, affecting at least 15% of them, with almost half of the cases attributed to male factors [1]. The causes of male infertility vary, with idiopathic causes (28.4%) and varicocele (18.1%) being the most prevalent, followed by genital tract infections accounting for 15% of cases [2]. Furthermore, various environmental pollutants, such as heavy metals, PM2.5, endocrine-disrupting compounds, and microplastics—the latter recently detected in semen—may affect sperm quality in humans through mechanisms of action not fully elucidated [3].

Initially, it was believed that the presence of bacteria in semen directly indicated an infection; however, this hypothesis was not validated until the development of next-generation sequencing methods. Recent advances in techniques have shown that human semen harbors specific microbiota that can also contribute to sperm health [4]. Gimenes et al. reported that multiple microorganisms, including bacteria, viruses, and protozoa associated with sexually transmitted infections (STIs), were found in the semen of both asymptomatic and symptomatic individuals [5]. The most frequent causes of STIs of the seminal tract are *Chlamydia trachomatis*, *Mycoplasma species* (spp.), and *Ureaplasma urealyticum*, leading to inflammatory conditions such as urethritis, prostatitis, orchitis, and epididymitis, either symptomatically or asymptomatically [6]. Prostatitis and epididymitis can result in fibrosis, obstruction of the seminal vesicles, and disruption of the seminal microenvironment, thus affecting semen parameters. From epithelial cells of the genital tract, microorganisms can directly spread into semen. These pathogens may affect sperm vitality, leading to infertility through various mechanisms. One main mechanism is oxidative stress due to reactive oxygen species (ROS) released by a high number of inflammatory cells in semen (leucocytospermia). ROS damage sperm cell membranes, leading to DNA fragmentation and changes in sperm morphology. Oxidative stress or the direct effects of pathogenic microorganisms can also trigger apoptosis in sperm cells [7].

The aforementioned common sexually transmitted bacteria (STB) are found at a higher prevalence in infertile men [4,8] and women [9]. Additionally, the presence of *Ureaplasma urealyticum* and/or *Mycoplasma hominis* has been linked to in vitro fertilization (IVF) failure when detected in either partner’s sample [10].

HPV is another very common STI and is often asymptomatic. At least 200 HPV subtypes have been identified, including LR HPV and HR HPV subtypes. LR HPV subtypes are associated with benign lesions, such as skin warts and condylomata acuminata, as well as respiratory papillomatosis in children. In contrast, HR HPV subtypes are the primary cause of malignant neoplasms in the genital system, including cervical, vaginal, vulvar, and penile cancers, as well as oropharyngeal and anal cancers in both sexes [11].

Many studies have investigated the impact of HPV on various sperm parameters, often yielding contradictory results. However, overall evidence suggests that HPV infection can negatively affect sperm characteristics such as DNA integrity, motility, count, viability, and morphology. Additionally, it may induce the production of anti-sperm antibodies, thereby impairing male fertility [12,13].

STB infections alone are associated with chronic inflammation and infertility. Simultaneous HPV and STB infections may augment the harmful effects of HPV on human health. The latter issue has been studied more extensively in women. There is evidence that changes in normal vaginal flora, such as bacterial vaginosis and co-infections with STB, play a significant role in the persistence of HPV and cervical carcinogenesis [14,15,16,17,18].

In healthy fertile men, HPV has also been shown to be associated with altered semen microbiota, but not with STB [19]. HPV and STB co-infections have been investigated in men, mostly in urine, urethral, or anal samples, focusing on their potential effects on the genital tract and anal oncogenesis [20,21].

Recently, there has been increasing interest in research on HPV and STB co-infections in semen and their role in male infertility. However, the available literature on this topic remains limited. Olivera et al. reported a high prevalence of HPV infection among male partners of infertile couples in Argentina [22,23]. The authors found that co-infection of HPV with *Chlamydia trachomatis* leads to inflammation in semen and decreases sperm quality, which is not observed with HPV infection alone. Similarly, another study examining the semen of asymptomatic infertile men in Mexico indicated that HR HPV subtypes or co-infection of HPV with *Chlamydia trachomatis* can negatively impact sperm quality by inducing a pro-inflammatory state and oxidative stress [24]. Additionally, Fan et al. reported that HPV infection resulted in reduced sperm volume and total sperm count in semen samples from male partners of infertile couples in China [25]. They also noted that co-infection of HPV with *Ureaplasma urealyticum* adversely affected sperm motility and viability.

To our knowledge, no similar study has been conducted in the Greek population. This study aims to investigate the presence of single and co-infections of HPV subtypes and STB (*Chlamydia trachomatis*, *Ureaplasma* spp., and *Mycoplasma* spp.) in asymptomatic male partners of infertile couples and their possible impact on sperm parameters. Furthermore, we examine the effects of cryopreservation on these microorganisms to add practical knowledge useful for the operation of sperm banks and IVF procedures.

## 2. Materials and Methods

### 2.1. Study Group

We performed a cross-sectional study consisting of 82 asymptomatic, randomly selected, male partners of infertile couples, aged between 24 and 56 years, who were referred to the research lab of the Obstetrics and Gynecology clinic of the Faculty of Medicine, University of Thessaly, for semen analysis from January 2025 to May 2025. All participants in the current study provided written informed consent. Additionally, the participants were required to be partners of infertile couples, aged between 20 and 60 years, and able to provide a semen sample after a standardized period of at least 2–3 days of sexual abstinence. Participants with any known diagnosed genetic abnormalities or medical interventions like a history of vasectomy, recent chemotherapy or radiation, antibiotic use within three months, or failure to provide written informed consent were excluded from the study. The study was conducted in accordance with the Declaration of Helsinki and received approval from the Committee of the Faculty of Medicine, School of Health Sciences, University of Thessaly, Greece (protocol number: 6th/21 October 2024).

### 2.2. Semen Collection and Processing

Participants were instructed to urinate and thoroughly cleanse their hands and penis before semen sample collection to avoid possible contamination from the urine or external genitalia. A total of 82 semen samples were initially collected within 48 to 72 h of sexual abstinence and obtained in sterile polypropylene containers. Each fresh semen sample, with a minimum volume of 1.0 mL, was evaluated and divided into two equal aliquots. One aliquot was processed immediately to check for the presence of HPV and STB. At the same time, the other aliquot was plunged into liquid nitrogen (−196 °C) for later processing (three months post-cryopreservation) after adding an equal volume of cryoprotectant solution (Sidney IVF Sperm Cryopreservation Buffer, COOK Medical).

Semen analysis was conducted on fresh semen aliquots following the World Health Organization Guidelines (World Health Organization, 2021) [26]. Sperm cells were separated from seminal plasma by centrifugation at 2000 rpm for 5 min. The resulting pellet was washed twice with 1X PBS (phosphate-buffered saline, Thermo Fisher Scientific, Waltham, MA, USA). Genomic DNA extraction was carried out using the Macherey-Nagel Nucleo Spin Tissue kit (GmbH & Co., Berlin, Germany) in accordance with the manufacturer’s instructions. The purity and concentration of DNA samples, from both fresh and thawed sperm aliquots, were assessed using a Nano Drop 2000 spectrophotometer (Thermo Fisher Scientific) by measuring the A260/A280 ratio. The concentration of the extracted DNA ranged from 40 to 180 ng/μL. The integrity of the extracted DNA was evaluated by electrophoresis on a 1% *w*/*v* agarose gel. Of the 82 semen samples, 80 (97.56%) were further analyzed for HPV and STB; 2 (2.44%) sperm samples demonstrated poor DNA quality and were therefore excluded.

### 2.3. HPV and Sexually Transmitted Pathogen Detection

HPV amplification and genotyping were performed with the AmoyDx^TM^ Human Papillomavirus (HPV) Genotyping Detection Kit (Amoy Diagnostics Co., Ltd., Xiamen, China) on Rotor-Gene TM 6000 (Corbett Life Science, Sydney, Australia), as previously described [27].

Sperm samples were also examined for common STB, including *Chlamydia trachomatis*, *Ureaplasma* spp. (*parvum* and *urealyticum*), *Mycoplasma genitalium*, and *Mycoplasma hominis*. A multiplex CE-IVD polymerase chain reaction (PCR) detection kit (Sacace *C. trachomatis*/*Ureaplasma* spp./*M. genitalium*/*M. hominis *Real-TM PCR kit) was used to amplify specific genomic target regions of the cryptic plasmid, Ure C, gyr B gene, and 16s rRNA gene, respectively. All PCRs were prepared according to the manufacturer’s protocol and conducted on a real-time PCR Rotor-Gene^TM^ 6000 (Corbett Life Science). An internal control (IC) was also added to each sample during DNA isolation to assess the extraction procedure and identify any potential reaction inhibition. The PCR profile was as follows: an initial hold at 95 °C for 15 min, five cycles of denaturation at 95 °C for 5 s, annealing at 60 °C for 20 s, and extension at 72 °C for 15 s, followed by 40 additional cycles of denaturation at 95 °C for 5 s, annealing at 60 °C for 20 s (during which fluorescence was measured), and extension at 72 °C for 15 s. To ensure the accuracy of all steps, negative controls for extraction and amplification, as well as a positive control, were included in each run. The total volume of each reaction was 25 μL, and for all samples tested, a starting concentration of 100 ng per reaction was used. Results were interpreted based on whether fluorescence curves crossed the threshold line. The boundary value of the cycle threshold (Ct) was based on the positive control Ct values as follows: FAM < 30 and <33 for all other channels (JOE/HEX, ROX, Cy5, and Cy5.5). A sample was considered positive for one or more channels if the fluorescence curve(s) crossed the threshold line.

All fresh semen aliquots were systematically tested for the presence of both HPV types and STB. We thawed and examined the frozen sperm samples at three-month intervals in cases that tested positive for either HPV subtypes or STB to determine if the viral or bacterial load was maintained after the freezing process.

### 2.4. Statistical Analysis

Statistical analysis was performed using SPSS v22. Data were expressed as mean + standard error of the mean (SEM). Comparison between HPV/STB-positive and HPV/STB-negative groups was performed using the non-parametric Mann–Whitney test whenever appropriate. The correlation between the parameters was also analyzed using the Spearman correlation test. The level of statistical significance was set at less than or equal to 0.05 (*p* ≤ 0.05).

## 3. Results

In this cross-sectional study, we successfully analyzed sperm samples from 80 men who were partners of infertile couples attending an infertility clinic for semen analysis. While the basic sperm parameters were within normal limits, the average percentage of sperm with normal morphology was at the borderline level of 4%, according to the Kruger criteria (WHO 2021) [28] (Table 1).

**Figure 1:**
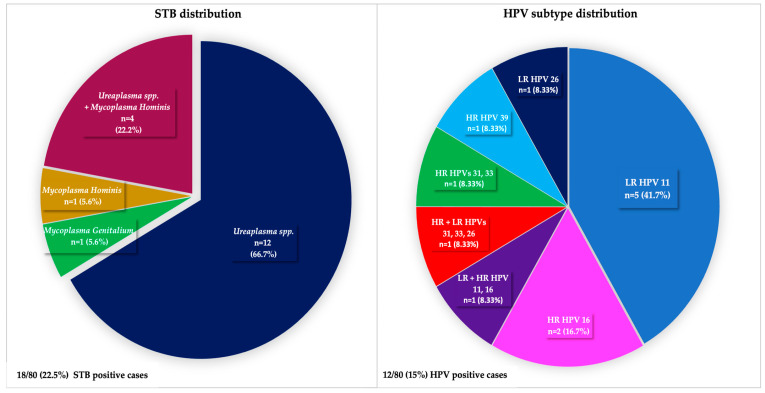
Distribution of STB and HPV subtypes among the positive cases. The charts illustrate the relative prevalence of specific pathogens. On the left chart, *Ureaplasma* spp. is the most frequent, followed by co-infections of *Ureaplasma* and *Mycoplasma hominis*. In the right chart, among HPV subtypes, LR HPV 11 is predominant, whereas among HR HPV subtypes, HPV 16 is predominant.

Of the 80 samples tested, 18 (22.5%) were positive for STB, and 12 (15%) were positive for HPV, including both HR and LR types (Figure 1). Importantly, no sample tested positive for both STB and HPV simultaneously. *Ureaplasma* spp. were the most commonly identified organisms, present in 16 out of the 18 positive cases (88.89%). Additionally, four of these *Ureaplasma*-positive cases (out of 16, 25%) showed co-infection with *Mycoplasma hominis*, which was the second most common pathogen in our study (5 out of 18, 27.78%). In the HPV-positive samples, both HR and LR HPV types were identified, with HPV 16 being the most prevalent among the HR types. Co-infection with both HR and LR types occurred in two cases (2/12, or 16.67%).

The samples that were positive for STB showed statistically significantly higher sperm concentrations and a greater percentage of sperm with progressive motility (Table 2).

Table 3 shows the comparison of sperm parameters between HPV-negative and HPV-positive samples. It is noted that the HPV-positive group exhibited significantly lower sperm volume and total sperm count.

In the thawed samples (30 positive samples in total; 18 STB and 12 HPV infections) after three months of cryopreservation, LR HPV types and STB were undetectable, whereas HR HPV types remained detectable.

## 4. Discussion

The current study investigates the effects of the most common STB and HPV types on sperm parameters. This cross-sectional study involved 80 sperm samples from asymptomatic men in infertile couples who were seeking sperm analysis. Among these samples, 22.5% tested positive for STB, while 15% were positive for HR and LR HPV types. Notably, no sample harbored an STB and HPV co-infection.

### 4.1. STB and Sperm Parameters

*Ureaplasma* spp. *(Parvum* and *Urealyticum* species) were the most common, followed by *Mycoplasma hominis*. *Chlamydia trachomatis* was not found in any of our samples. According to data from the European Center for Disease Prevention and Control (ECDC, 2024) [29], Chlamydia infections are decreasing but remain the most prevalent STB in Europe. However, studies in the Greek population report prevalence rates that differ from those in other European countries. A study by Chra et al. [30] on high-risk individuals with STB in Greece reported a prevalence of 5.7% in symptomatic males, while only one asymptomatic male was infected. A study by Ikonomidis et al. [31], conducted in Central Greece, showed a low prevalence of *Chlamydia trachomatis* and *Mycoplasma* species (*Genitalium* and *Hominis*). Nevertheless, a high prevalence of *Ureaplasma* spp. was observed in both sexes. In our study, 16 out of 80 cases (20%) tested positive for *Ureaplasma* spp., and 5 out of 80 cases (6.25%) were positive for *Mycoplasma hominis*, with 4 of these cases being co-infections. Another study by the same research group in Central Greece found that *Ureaplasma* spp. was the most common STB infection, followed by *Mycoplasma hominis*. This study also noted that the total sperm count and motility were unaffected by the presence of STB in Greek males undergoing semen analysis [32]. These findings align with our results.

Previous studies have revealed that certain STB may affect sperm parameters; however, their influence remains under investigation, as conflicting data and differences between infertile and fertile men have been reported. In our study, STB-positive samples exhibited higher sperm concentrations and significantly improved progressive motility compared with negative cases (Table 2).

A recent study by Potiris et al. indicated that pathogens such as *Ureaplasma*, *Mycoplasma*, *Chlamydia trachomatis*, and *Neisseria gonorrhoeae* may provoke immune responses by producing reactive oxygen species (ROS) and pro-inflammatory cytokines, leading to sperm damage—particularly if inflammation is persistent and untreated [33]. These pro-inflammatory cytokines seem to influence oxidative stress and sperm metabolism. In the study by Reichart et al. (2001), it was proposed that when sperm motility relies on mitochondrial oxidative phosphorylation, this usually occurs at low pH (pH < 7) [34]. Under these conditions, *Ureaplasma urealyticum* competes with mitochondrial respiration in sperm cells, resulting in decreased sperm motility and viability. Conversely, at higher pH values, *Ureaplasma urealyticum* stimulates glycolysis, which increases sperm motility. Although our study lacks inflammatory markers and biochemical data, the variable effects of *Ureaplasma urealyticum* on sperm metabolism may further explain our findings.

### 4.2. HPV and Sperm Parameters

HPV is recognized as the most common sexually transmitted infection. Its prevalence varies between fertile and infertile males, ranging from 2% to 31% in fertile and from 10% to 35.7% in infertile individuals [35]. In our study, HPV-positive samples accounted for 15% of the cases examined, which aligns with previously reported data indicating a prevalence of 15.2% in the European population [36]. Additionally, HPV-positive samples exhibited lower sperm volume and increased non-progressive sperm motility. HR HPV 16 subtype was more prevalent, in agreement with findings from other studies [19,27]. The impact of HPV infection on sperm parameters has been extensively studied, although the findings are controversial. Recently, Francis et al. [37] investigated the association between HPV infection and semen parameters in 246 men, reporting higher total and progressive motility in the non-male infertility group of infertile couples.

Sperm volume was significantly lower in samples that tested positive for HPV compared to those that were HPV-negative. Several studies have established a link between HPV infection and a decrease in sperm volume. Our findings align with the research conducted by Damke et al. [38], who analyzed 229 semen samples and suggested that HPV infection in semen is associated with decreased sperm volume, abnormal viscosity, and increased seminal pH values. Conversely, a meta-analysis by Weinberg et al. [39] did not show a definitive effect of HPV infection on semen volume. Oligospermia could be linked to the impact of HPV on prostate gland function, as the prostate plays a key role in producing sperm plasma. HPV may reduce the prostatic secretions either directly or indirectly, affecting its ability to secrete sperm plasma. This difference likely results in a lower total sperm count in HPV-positive samples, as reflected in the variation in sperm volume between the two groups. Males with HPV present in their semen exhibit glandular dysfunction and altered proportions of seminal and prostate vesicles. This may be related to zinc production issues, which are regulated by testosterone and can lead to inadequate sperm condensation [38,40]. In addition to the previous suggestions, the younger age observed in the HPV-negative group may also contribute to the interpretation of the results.

### 4.3. STB-HPV Co-Infection

An interesting finding of this study is the absence of co-infection of STB and HPV in all tested sperm samples. Previous research has reported a significant incidence of co-infection between *Chlamydia trachomatis* and various HPV types. For instance, a study by Olivera et al. [23] found a co-infection between HPV and STB in more than 70% of cases, with HPV-*Chlamydia trachomatis* being the most common co-infection (52% of cases). In another study conducted by Gimenes et al. [41], 76 men were enrolled, and 42 out of 52 asymptomatic males showed a sexually transmitted infection. The most frequent co-infections were also HPV with *Chlamydia trachomatis,* as well as HPV with *Trichomonas vaginalis,* with similar numbers estimated (3/76 total cases, 7.1% of 42 ST-positive cases for both co-infections).

In another study conducted by Rivera et al. (2022) among 81 asymptomatic males without urogenital infection symptoms, 78/81 (96.3%) of the collected semen samples were positive for at least one pathogen [42]. Interestingly, 23 samples were positive for *Chlamydia trachomatis*, while HPV DNA was not detected in any of the examined samples. Additionally, Olivera et al. (2021), in another study conducted on 117 male partners of infertile couples, presented 20/117 (17.1%) males with an HPV-STB co-infection and showed a significant association between HPV and *Chlamydia trachomatis* co-infections, although this was based on a low number of *Chlamydia trachomatis* positive samples, highlighting the importance of screening for urogenital infections in the initial diagnostic procedures of infertile couples [22].

In the current study, none of the sperm samples tested positive for the presence of *Chlamydia trachomatis*. These findings may be influenced by the geographical context of the research, which was conducted in Central Greece. Previous studies indicate that the prevalence of common STIs varies significantly across different regions, countries, and demographic groups. Factors such as age, ethnicity, and socioeconomic status play a critical role in this variation [43]. Another possible reason for the lack of *Chlamydia trachomatis* infection and co-infections among the sperm samples could be that they were collected from male partners of infertile couples. However, this does not necessarily mean that these men themselves were infertile. *Chlamydia trachomatis* infection is strongly associated with male infertility, so the absence of this infection in these men may be related to their reproductive health status. Unfortunately, data on co-infections involving HPV and STB are limited, highlighting the need for further studies, including studies involving larger populations.

### 4.4. STB, HPV, and Cryopreservation

Our study revealed an interesting aspect of HPV persistence following cryopreservation. HR HPV types remained detectable even after 3 months of cryopreservation, whereas LR HPV types were no longer detectable during the same period. A study conducted by Foresta et al. [44] also noted the persistence of HPV infection in semen samples, despite various washing procedures. This difference in persistence may be attributed to the distinct mechanisms of action of these virus types. One hypothesis is that HR HPV types may integrate into sperm DNA, making them resistant to removal during the washing procedures typically used in cryopreservation. In contrast, LR HPV types have a low oncogenic potential and possibly exist primarily as episomes or extracellular vesicles. They tend to remain on the surface of sperm and may be removed during washing. This notion is in agreement with the study of Capra et al., who found that semen infections with at least one HR HPV type showed a substantial increase in genomic damage compared to LR-only infections [12].

HR HPV subtypes’ persistence after cryopreservation also raises the issue of routine HPV screening of donor sperm samples in sperm banks. This is not only because intrauterine inseminations and assisted reproduction techniques using HPV-positive semen may result in fewer pregnancies and higher miscarriage rates but also because of concerns regarding the safety of females, who can be infected with HR HPV subtypes after sperm insemination [39,45,46].

In contrast to HR HPV infections, neither *Mycoplasma* nor *Ureaplasma* spp. was detectable in thawed samples. and LR HPV types were also not detectable. *Mycoplasma* and *Ureaplasma* bacteria lack cell walls, have small genomes, and can survive outside cells. They replicate through processes such as binary fission or budding [47,48]. Unlike viruses that integrate their genomes into the haploid DNA of sperm, these bacteria do not integrate their genetic material and, typically, are attached to the head and midpiece of sperm cells [49].

In this study, we present preliminary data on the presence of STB and HPV infections in Greek asymptomatic male partners of infertile couples and their effect on sperm parameters. Additionally, we studied the impact of cryopreservation on these microorganisms to provide practical insights for the operation of sperm banks and IVF procedures. However, our study has limitations concerning the sample size, which was restricted to a specific geographic area. Although the sample size is small, conclusions can be drawn. Furthermore, the present study lacks a control group and data on lifestyle factors or inflammatory markers [50].

Future research with a larger sample size should be performed to clarify the mechanisms and pathways through which these pathogens may act pre- and post-cryopreservation. To this end, additional methods (e.g., fluorescent in situ hybridization (FISH), detection of viral oncogene transcripts, integration assays) should be used to confirm the impact of integration in the host genome.

## 5. Conclusions

In conclusion, to the best of our knowledge, this is the first study in a Greek population examining HPV subtypes simultaneously with common STB in semen before and after cryopreservation. Slight differences regarding sperm volume, concentration, PRM, and NPM were observed between the STI-positive and negative groups. Interestingly, a co-infection of STB and HPV was not found. HR HPV remained detectable even after three months of cryopreservation, whereas LR HPV and STB were not detectable during the same time period. These preliminary findings are interesting, as they could be useful for routine screening for HPV and STB in sperm samples preserved in sperm banks.

## Figures and Tables

**Table 1:** Basic sperm parameters in specimens derived from 80 participants.

Number of Cases	80
Age (years)	38.7 ± 0.8
BMI (Kg/m^2^)	27.03 ± 0.4
Abstinence (days)	3.7 ± 0.1
Volume (mL)	3.4 ± 0.2
Concentrations (mil/mL)	39.01 ± 3.07
Total number (mil)	124.5 ± 12.7
PRM (%)	43.05 ± 2.1
NPM (%)	10.45 ± 0.9
IM (%)	41.1 ± 2.4
Morphology (%)	3.9 ± 0.7
TZI	1.5 ± 0.09

BMI: body mass index, PRM: progressive motility, NPM: non-progressive motility, IM: immobility, TZI: teratozoospermia index.

**Table 2:** Comparison of sperm parameters in positive and negative samples for STB.

Number of Cases	62/80 Negative (77.5%)	18/80 Positive (22.5%)	*p* Value (<0.05)
Age (years)	38.4 ± 0.9	39.7 ± 1.8	NS
BMI (Kg/m^2^)	26.8 ± 0.4	27.5 ± 0.5	NS
Abstinence (days)	3.6 ± 0.1	3.8 ± 0.3	NS
Volume (mL)	3.5 ± 0.2	2.9 ± 0.2	NS
Concentration (mil/mL)	35.7 ± 3.5	50.3 ± 5.8	<0.05
Total number (mil)	117.4 ± 14.6	154 ± 23.4	NS
PRM (%)	39.9 ± 2.3	53.6 ± 3.6	<0.05
NPM (%)	10.2 ± 1.1	11.2 ± 1.6	NS
IM (%)	42.8 ± 2.8	35 ± 4.6	NS
Morphology (%)	3.6 ± 0.7	5.1 ± 2.3	NS
TZI	1.5 ± 0.1	1.7 ± 0.06	NS

BMI: body mass index, PRM: progressive motility, NPM: non-progressive motility, IM: immobility, TZI: teratozoospermia index, NS: non-significant.

**Table 3:** Comparison of sperm parameters in negative and positive samples for LR and HR HPV types.

Number of Cases	68/80 Negative (85%)	12/80 Positive (15%)	*p* Value (<0.05)
Age (years)	38.1 ± 0.9	42.7 ± 1.8	<0.05
BMI (Kg/m^2^)	26.8 ± 0.3	28.3 ± 1.6	NS
Abstinence (days)	3.7 ± 0.1	4 ± 0	NS
Volume (mL)	3.5 ± 0.2	2.7 ± 0.3	<0.05
Concentrations (mil/mL)	40.3 ± 3.4	31.3 ± 6.4	NS
Total number (mil)	130.9 ± 14.3	84.5 ± 17.7	<0.05
PRM (%)	42.6 ± 2.3	45.5 ± 4.5	NS
NPM (%)	9.3 ± 0.9	16.5 ± 2.6	<0.05
IM (%)	41.6 ± 2.7	38.1 ± 5.1	NS
Morphology (%)	3.9 ± 0.7	4 ± 0	NS
TZI	1.5 ± 0.09	1.9 ± 0	NS

BMI: body mass index, PRM: progressive motility, NPM: non-progressive motility, IM: immobility, TZI: teratozoospermia index, NS: non-significant.

## Data Availability

The original contributions presented in this study are included in the article. Further inquiries can be directed to the corresponding author.

## Abbreviations

BMI	Body Mass Index
ECDC	European Center for Disease Prevention and Control
HPV	Human Papillomavirus
HR	High Risk
IM	Immobility
IVF	In Vitro Fertilization
LR	Low Risk
NPM	Non-Progressive Motility
NS	Non-significant
PCR	Polymerase Chain Reaction
PRM	Progressive Motility
ROS	Reactive Oxygen Species
SEM	Standard Error of the Mean
STB	Sexually Transmitted Bacteria
STI	Sexually Transmitted Infection
TZI	Teratozoospermia Index
